# Pharmacotherapy literacy level and predictors of low literacy among diabetes mellitus type 2 patients in Serbia

**DOI:** 10.1186/s12889-023-16639-y

**Published:** 2023-09-19

**Authors:** Marija Levic, Natasa Bogavac-Stanojevic, Stana Ubavic, Dusanka Krajnovic

**Affiliations:** 1 Doctoral Program of Department of Social Pharmacy and Pharmaceutical Legislation, Faculty of Pharmacy, Univesrity of Belgrade, 11221 Belgrade, Serbia; 2 Department of Medical Biochemistry, Faculty of Pharmacy, University of Belgrade, 11221 Belgrade, Serbia; 3 Medicines and Medical Devices Agency of Serbia (ALIMS), 11221 Belgrade, Serbia; 4 Department of Social Pharmacy and Pharmaceutical Legislation, Faculty of Pharmacy, University of Belgrade, 11221 Belgrade, Serbia

**Keywords:** Literacy related to medicines, Patients, Diabetes mellitus type 2, Self-reported instruments, Self-management

## Abstract

**Background:**

Pharmacotherapy literacy (PTHL) is a specific ability to safely access, appraise and understand the available information concerning medication and to calculate and act accordingly. The concept of PTHL is mostly unknown for the majority of diabetes mellitus type 2 (DMT2) patients in Serbia. With diabetes being one of the major public health problems in Serbia with a prevalence of 9.1%, this two-study research aims at constructing performance-based instrument and estimating the prevalence of PTHL levels and identification of predictors of low PTHL scores in patients with DMT2.

**Methods:**

Multistage study was performed to adapt the existing performance–based instrument (PTHL-SR) into specific questionnaire for DMT2 population (PTHL-DM instrument). PTHL levels were assessed through cross-sectional study categorising patients into groups of low, medium, and high PTHL levels. We considered 19 predictors for low PTHL scores, from sociodemographic characteristics, health behaviours and health characteristics, access to health-related information and empowerment-related indicators. Univariate and multivariate logistic regression analyses were used to determine independent predictors of low PTHL.

**Results:**

The final 15-item PTHL-DM instrument proved to have satisfactory reliability (KR20 = 0.475) and internal reliability [ICC for the whole instrument was 0.97 with 95% confidence intervals (0.95–0.99)]. Positive correlation (rho = 0.69) between PTHL-DM score (15 questions) and the total PTHL-SR score (14 questions) was also observed.

It was demonstrated that the majority of 350 patients had low PTHL (62%), and only 5% high PTHL level. Mean score on PTHL-DM was 7.8 ± 2.3. Probability of low PTHL increased among smokers, patients with low interest in health and those who estimated their health as bad. Patients who used pharmacists as sourse of information were less likely to be pharmacotherapy illiterate. Combined therapy with insulin and Oral Hypoglycemic Agents was associated with higher PTHL.

**Conclusions:**

Our data indicate that specific PTHL-DM tool is objective, valid, and reliable. It was found that low level of PTHL prevailed among DMT2 patients. Medication literacy is influenced by age, residence, education, and family status. Patients with better health literacy also reported better health behaviours. Different patient empowerment programs and approaches aimed at raising PTHL would be essential to improve self-management and control of this widespread chronic disease in Serbia.

**Supplementary Information:**

The online version contains supplementary material available at 10.1186/s12889-023-16639-y.

## Background

An adequate level of pharmacotherapy literacy (PTHL) is extremely important for chronic patients in order to properly use their prescribed therapy [1, 2], reduce the number of adverse events, improve medication adherence [3], and prevent hospitalisations [4].

These patients have to continuously implement different activities for proper management of their therapy. The concept of health literacy (HL) is of growing importance in public health and the healthcare system. Possession of appropriate knowledge and skills contributes to the prevention of diseases, improvement of health and quality of life. The concept in itself is insufficient for chronic patients who require certain knowledge about the use of medicines to control their disease. Therefore, a new term was required that would deal with literacy related to the use of medicines. In the relevant literature there are several names proposed, such as: "medication literacy" [5] "medical literacy" [3, 6], and "pharmacotherapy literacy" [7]. The most widely used definition of PTHL implies: "The individual’s capacity to find, evaluate, calculate, and understand reliable information related to pharmacotherapy and pharmacy-related services that is needed to make appropriate medication-related decisions, regardless of the method of transmission and the content of the information (written, spoken information, image or symbol), and thereby reduced the risk of bad outcomes of pharmacotherapy" [8, 9]. This definition was modified by Pantuzza et al. (2022) by adding the term "assess" and "digital information" so that the latest definition reads: "The degree to which individuals can obtain, understand, communicate, calculate and evaluate patient-specific information about their medications to make informed medicin and health decisions, to use their medications safely and effectively, regardless of how the content is delivered (eg written, oral, digital and visual)". As pharmacotherapy literacy is derived from health literacy specifically targeting medication-related skills, Pentuzza et al., adopted the definitions of functional, communicative, and critical literacy known from Nutbeam [10], and the concept of numeracy proposed by Golbeck et al. (2005) [11]. Hence, the suggested conceptual model identified specific components for literacy in the context of medication use and in this way expands known HL's concepts capturing broad skills that influence medication use [12]. It encompasses dimensions of functional literacy, communicative literacy, critical literacy, and numeracy with its respective subdimensions (understand, access, communicate, evaluate, calculate)smedication-related information [12].

Patients who have a lower level of PTHL are more susceptible to medication addiction. Recognising such patients in everyday practice is difficult. For this purpose, a set of questions was developed in the form of a questionnaire—Recognition and treatment of patients with low pharmacotherapeutic awareness (RALPH). High health awareness is crucial for patients to be able to understand information and instructions related to their medical treatment. A significant number of patients do not have a sufficient level of health awareness and literacy. Such patients face difficulties in interpreting the information related to the prescribed medicine. The timely identification of such patients with potentially low pharmacological awareness, which we will call PTHL, is certainly important, given that those patients may be at increased risk of medication addiction. Previous research has shown that pharmaceutical staff primarily use their gut feeling or certain patient characteristics to identify patients with lower health literacy. The RALPH method presents a questionnaire as a practical tool for identifying those patients. The results showed that most patients with diabetes have sufficient knowledge about how often and when they should take their medicines, but also that they are more prone to encountering problems in more complex tasks such as interpreting warnings and precautions and critically analysing the information obtained about the medicine [4]. Identifying available and effective methods to improve PTHL among patients is one of 20 research priorities, highlighted in a study that used an inclusive, systematic, and replicated process to define medication safety research priorities [13]. In this regard, the World Health Organization (WHO) launched the third WHO Global Patient Safety Challenge, “Medication Without Harm”, in 2017, which seeks to facilitate a series of strategic initiatives aimed at improving medication safety worldwide [14].

### PTHL in people with diabetes mellitus type 2

DM is one of the chronic diseases that require high PHTL levels because patients self-regulate their dose of therapy on a daily basis.

It was found that despite advancedtherapy and the availability of different guidelines for clinical practice, only about 30% of patients with DM manage to achieve target glycemia and blood pressure values [15]. An appropriate PTHL level will help with their awareness of therapy and health behaviour.

Patients with DM and low PTHL have different issues with understanding of instructions, different health advices and warnings. These patients also have poorer communication with healthcare professionals [16, 17]. For this reason, it is very important to assess their PTHL level and implement appropriate training in order to improve control over the disease itself. Numerous interventions in upgrading education have shown good results in patients with low PTHL and improved diabetes outcomes [18, 19].

### Predictors associated with HL and PTHL

Sociodemographic factors, lifestyle and environment, may interfere an individual's skills and knowledge about PTHL. It was confirmed that education level, age, and income level can influence making the right health decisions [20–22].

Some research indicates that HL can help prevent health inequalities marginalised populations [21–25].

The objectives of the research are to adapt the existing performance-based instrument currently used in the Serbian language in relation to medicines, making it specific to DMT2 patients and to identify its validity and reliability before using it for the assessment of PTHL and identification of predictors of low PTHL.

## Methods

### Research studies

We conducted two-study research: first, we constructed the instrument (formatted and adapted the existing instrument [7] and gathered data for its psychometric properties) and then we applied it. As we needed a specific and performance-based instrument in the Serbian language to be able to assess PTHL in DMT2 patients, in the first study, we adapted pre-existing self-reported objective medication instrument in Serbian (PTHL-SR), [7, 9] to make it specific to the DMT2 population (PTHL-DM). A cross-sectional study design was adopted for the data collection, first for validation of the measuring instrument between January 2021 and June 2021 and then for the evaluation study during December 2021 and between March and May 2022.

The target population of this research for both studies were patients diagnosed with DMT2 at least six months before the start of the study, who knew the Serbian language, aged 18 and older and voluntarily agreed to participate with signed informed consent. The exclusion criteria were participants with medical backgrounds (e.g., doctors, study nurses, and those with blindness, dementia, or psychotic disorder). The data collection was carried out at one Community Healthcare Center in the municipality of Zvezdara and one Community pharmacy from the municipality of Vozdovac. Both municipalities are located in Belgrade, the capital of Serbia. Users of internal medicine services in Belgrade are mostly middle-aged or elderly. In 2020, the scope of work of internal medicine services in the Healthcare Center "Zvezdara" included 24,287 examinations, where the daily overload was slightly more than 19 examinations. This is almost 10% of all internal medicine examinations performed that year in all municipalities of Belgrade [26]. This is a typical primary healthcare institution for those patients, and it fully represented a demographically diverse population. The adequate age and gender distribution of the sample reflected the targeted population well and could be considered representative of elderly people of DMT2 in the country [27]. According to the 2022 census, Zvezdara has 171,278 inhabitants, of which 80,084 are men and 91,194 are women, or 10% of the population of Belgrade and approximately 3% of the population of Serbia. Zvezdara includes 4 municipalities, the largest that is urban area and 3 suburban areas [28].

In Vozdovac there are 169,695 inhabitants, or 9% of the population of Belgrade and approximately 2.5% of the population of Serbia. It contains 36 settlements, from urban, suburban, and rural areas. These two municipalities were chosen for recruitment, since the highest proportion of persons are registered as permanent community, and both rural and urban areas are covered. Patients from all parts of those municipalities were represented to reflect the geographical distribution in the target population [29].

Residents of Belgrade account for a fifth of those who died from diabetes in Serbia. They predominantly suffer from type 2 diabetes, from which in 2021, 1,728 people (87.7% of the total number of new cases) fell ill. Of the total number of DMT2 patients registred in Belgrade (81.257 on December 31,2021), one third belonged to the population of 65 and over. In our sample this age group was slightly over represented (39.3%). The prevalence of diabetes is higher in males, with the difference between the sexes being the least present in older citizens in Belgrade who are 65 years and over [26, 30]. In our sample woman were prevalent, but man dominated in that age group (43.6%)**.** All these indicates that the sample reflected the elderly with DMT2 population in Belgrade well.

### Instrument construction and data collection in the first study

**Fig. 1:**
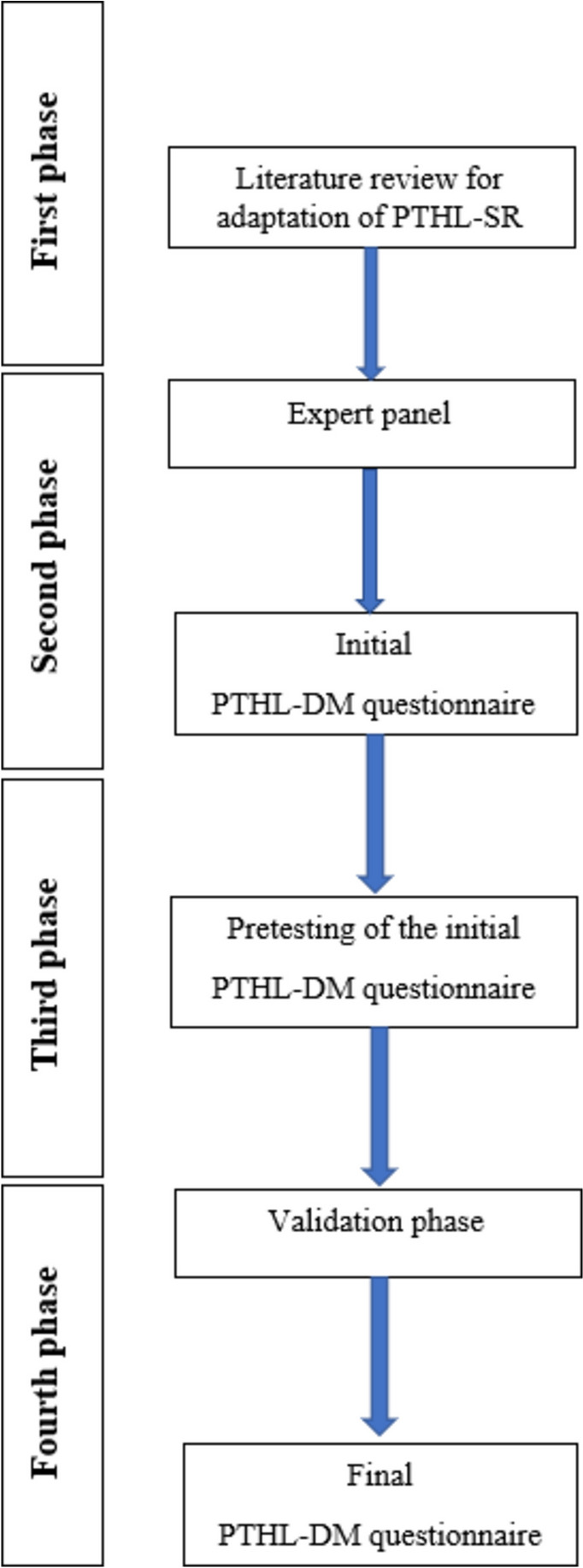
Schematic diagram of the construction of the PTHL-DM questionnaire

In the first study, a multi-phase procedure was carried out (Fig. 1) for the formation of the measuring instrument including: (1) Literature review to adapt the existing version of the PTHL-SR by rephrasing the items and adding some new ones to create the initial PTHL-DM, (2) an expert panel for face validity of the adopted version (initial PTHL-DM), (3) pre-testing of the initial version and (4) a study to collect validity evidence and formation of the final version.

- 1) A review of the PubMed database was performed in order to find literature that dealt with the examination of PTHL persons with DMT2 or the knowledge and understanding of information about medication and their use in this population. Key words for the literature search were: "pharmacotherapy literacy", "diabetes mellitus type 2", "information", "knowledge", and "medications". This process generated a list of potential items that could be included in the existing version of PTHL-SR. It contains 14 medication-specific questions, divided by domains based on Nutbeam's research [31, 32] distinguishing three types of HL: functional, interactive/communicative, and critical. The domains of initial PTHL contain three types of HL: 5 questions to assess knowledge about the use of medicines, 3 questions to assess the understanding of written and spoken information about the use of medicines, 5 questions to assess the numerical abilities required for adequate administration of correct doses of medicine and 1 question related to access to information about medicines. Secondly, the authors worked together to revise some of the existing questions of the PTHL-SR to be diabetes–specific and made an initial version of PTHL-DM. Based on the literature, the authors divided the questions according to the areas (domains) relevant to proper use of medications, [33, 34], as medication-related literacy includes skills not only for patients to have access to sufficient information but also sufficient reading, coding and self-management.
- 2) The content of the questionnaire (initial PTHL-DM) was defined using expert review [35–37]. The expert panel consisted of three professors from the Faculty of Pharmacy in Belgrade, two pharmacists working in practice experienced with pharmacy practice research, one endocrinologist, and one general practitioner. The team of experts were aimed with assessing the face validity and making informal assessments that yield open ended comments about the PTHL-DM items to be evaluated. They discussed the importance and relevance of each potential correction, adaptation or additional item and additionally discussed: (i) the question form and answer form, (ii) the suitability of the information in question, (iii) the clarity of the graphic drawings that accompanied certain questions and (iv) whether or not the question should be in the instrument. After expert panel, the adapted version of PTHL-DM was created for the pre-test phase.
- 3) Pre-testing was carried out by filling in the initial PTHL-DM instrument from 10 participants suffering from DMT2, who were not later included in the study. By pretesting the instrument, additional data were obtained on the clarity, transparency, and format of the adapted PTHL–DM instrument [38].

After adaptation, the initial version of PTHL–DM instrument was pre-tested on 10 interviewed persons to check whether the items were understandable and logical. The mean age of interviewed participants in the pre-test was 62.7 ± 12.4 years, ranging from 34 to 79 years of age, of which 60% were males. After the pretest, the results were discussed with the group of researchers. A version of the PTHL-DM instrument containing 15 questions divided into 4 PTHL groups based on their definition was agreed upon—questions about access to information (2 questions), understanding (3 questions), interpretation (4 questions) and use of information (6 questions).

The following is an explanation of each definition:

- Access to health information refers to the ability to search for, find and obtain information.
- Understanding means the ability to understand the information found.
- Interpretation describes the ability to reproduce, select and judge and evaluate found health information.
- The use of health information refers to the communication and use of information in order to make decisions that maintain or improve health.

Eleven out of 15 questions were medication specific with a variety of medication information represented (medication name, dosing information, treatment indication, precautions, time and prediction of therapeutic effect etc.). Hypothetical prescription labels were part of the instrument. Some of the questions included graphic presentations of a standardised measuring device for dosage of liquid medicines. There were also questions related to interpretation of the composition, nutritional values, and declaration. Two questions were related more to disease/general health literacy and were not medication specific. (Fig. 1).

- 4) For collecting validity evidence for the 15-items instrument, following the recommended ratio of at least 10 participants for each instrument item, the adequate sample size was 150, to which we added 10% to cover possible withdrawals [39]. Hence, we conducted a study on a convenient sample of 164 DMT2 patients. but 14 responders were excluded from the analysis due to not fulfilling 90% of the instrument.

Before this survey, we recruited five research assistants to help us with collecting data. To ensure that they were familiar with the purpose, process, and procedure of applying the instrument, we systematically trained three pharmacy graduates and two doctors as research assistants. The interviewers (researchers and assistants) explained the purpose and significance of the study to the participants and obtained written informed consent. Study participants were interviewed face-to-face for collection of sociodemographic data. The instrument applied in this validation study was self-administered in paper-and-pencil form. At any moment, at least one interviewer was present to provide all necessary explanation. Participants did not receive any payment for participation. All data was entered as anonymous into the database.

### Sample and data collection for the second study

The required number of participants was calculated based on the population of DM patients in the Belgrade (80.241) area. The share of adult residents aged 20 to 64 in the total population in Belgrade in 2020 was 60.2%, and the share of the population aged 65 and over in the total population was 20% [26]. For calculation, we used estimated percentage of high PTHL from the literature, which was 72,83% [41, 42], and 95% confidence interval with an error of 5%. Based on these parameters, the required sample was at least 353. The required sample size of 353 was increased by 10% due to potential dropouts (accounting for the non-responder rate) during the study. A total of 385 DMT2 patients were approached, of which 90% agreed to participate. The final sample consisted of 350 DMT2 participants. The instrument for PTHL (PTHL-DM) was filled out voluntarily and anonymously by the participants after they had received the necessary and detailed information from the interviewers about the aim and the course of the research. Glycemic control was assessed by the most recent HbA1c value in the patient`s medical record at doctors` office, or from the laboratory record that patient brought along with them to their pharmacy visits. Sociodemographic characteristics of participants included age, gender, marital status, children, education level, employment and income. Health characteristics and health behaviors` information included HbA1c value, therapy, frequency of therapy application, exercise, alcohol and smoking were related to. Access to health-related information (a primary source of information), and empowerment-related indicators (perceived interest in one’s health and perceived self-assessment of one’s health in general) were recorded as well [43, 44]. Health literacy was assessed the same way like PTHL, using the validated multidimensional perception-based instrument—FCCHL-SR12 instrument [45].

### Data analysis

To assess the inter-rater (test – retest) reliability or consistency among the observational ratings, we calculated Kappa coefficient for the dichotomous data. The Kappa coefficient value was defined by Altman [46]. Interclass correlation coefficient (ICC), kurtosis and skewness were calculated for the items in each domain and the whole instrument. Also, the relationship for the domains between these PTHL-SR and PTHL-DM was examined by Spearman correlation.

Data normality for continuous variables was tested by the Kolmogorov–Smirnov test. Non-normal continuous variables are described by median and inter-quartile range, while normally distributed variables are presented by mean and standard deviation.

Categorical variables are spresented by absolute and relative frequencies and the difference between groups of categorical variables was examined by the chi squared (χ2) test of independence.

The responses on the PTHL-DM instrument were dichotomised into correct response (given a value of 1) and incorrect response / the patients didn't know (given a value of 0). The values were then summed up and the total PTHL-DM scores were obtained. Additionally, for each patient, the percentage of correct answers was calculated. As described previously [9] respondents were categorised according to their levels of PTHL-DM into those with a low level of PTHL (up to 8 correct answers (< 60%)); medium level of PTHL (between 9 and 11 correct answers (60–80%)); and high level PTHL-DM (between 12 and 15 correct answers (> 80%)). Access to information, understanding, interpretation and use of information showed skewed distributions and differences between the PTHL-DM levels groups were compared by the Kruskal Wallis test. Age showed normal distribution and it was compared by ANOVA.

Sociodemographic characteristics, healthcharacteristics and health behaviors, access to health-related information, and empowerment-related indicators were used as predictors of low PHTL level in univariate logistic regression analyses. Multivariate logistic regression analyses was used to determine independent predictors of low PTHL. Overall, *p* values less than 0.05 were considered significant.

All analyses were performed using IBM SPSS Statistics for Windows, Version 27.0. Armonk, NY, USA: IBM Corp. It was conducted by Jamovi Statistical Software (Idaho State University).

We conducted this study following the recommendations of the STROBE checklist [47].

## Results

### First study: Validation phase

Firstly, all questions from the PTHL-DM were analysed for the KR calculation. The KR20 score was 0.475.

Mean scores for PTHL-DM domains and total PTHL-DM and their reliability parameters are presented in Table 1. Skewness for the domain Access to information showed outliers in a distribution, other domains and total PTHL showed slight asymmetry and the majority had a negative coefficient, which pulls the mean value towards a lower value. Apart from the domain Access to information, kurtosis for others was negative and indicated the small outliers in a distribution.

**Table 1:** Scores and reliability parameters for PTHL-DM domains and total PTHL-DM

	**Domain**				
	**Understanding**	**Access to information**	**Interpretation**	**The use of information**	**PTHL-DM**
N of questions	3	2	4	6	15
Xsr ± SD	1.2 ± 0.8	1.7 ± 0.5	1.8 ± 0.9	3.5 ± 1.6	8.2 ± 2.3
Skewness	0,2	-1.3	-0.2	-0.3	-0.1
Kurtosis	-0.7	0.8	-0.5	-0.3	-0.8
Correct answers (Min—Max)	0–3	0–2	0–4	0–6	3–13
ICC (95% CI)	0.98 (0.96–0.99)	0.58 (0.11–0.80)	0.93 (0.83–0.97)	0.95 (0.89–0.98)	0.97 (0.95–0.99)

To determine the instrument’s consistency in the repeatability dimension, the ICC for the whole instrument was calculated to be 0.97 with 95% confidence intervals (0.95–0.99). Kappa coefficient was 1.00 for questions number 2, 3, 4, 5, 7 and 14, it is in the range 0.94–0.99 for questions number 1, 6, 8, 9, 11 and 15, and there are questions with lower ICC (number 10 = -0.47, 12 = 0.58 and 13 = 0.83).

#### Correlation

The relationship between the total PTHL-DM score (15 questions) and the total PTHL-SR score (14 questions) was good (rho = 0.69). A good correlation is for domain understanding (0.74), while for domain knowledge/interpretation and use of information is somewhat weaker (0.43, 0.50, respectively).

### Second study: Measurement of pharmacotherapy literacy

In total, 350 patients participated in the second study. The average age of participants was 62 ± 10.5 years ranging from 31 to 82 years of age. The percentage of participants with 65 age and older was 40% (*n* = 139). The majority of respondents were female (55.4%), married (53.8%), and individuals with higher education (60.6%). The prevalence of males and females aged 65 and older was similar (43.6% in males and 36.6% in females, *p* = 0.184). According to therapy regimen, the majority of respondents (76%) were on diet and Oral Hypoglycemic Agents (OHAs).

#### Percentage of correct answers in investigated PHTL-DM instrument

**Fig. 2:**
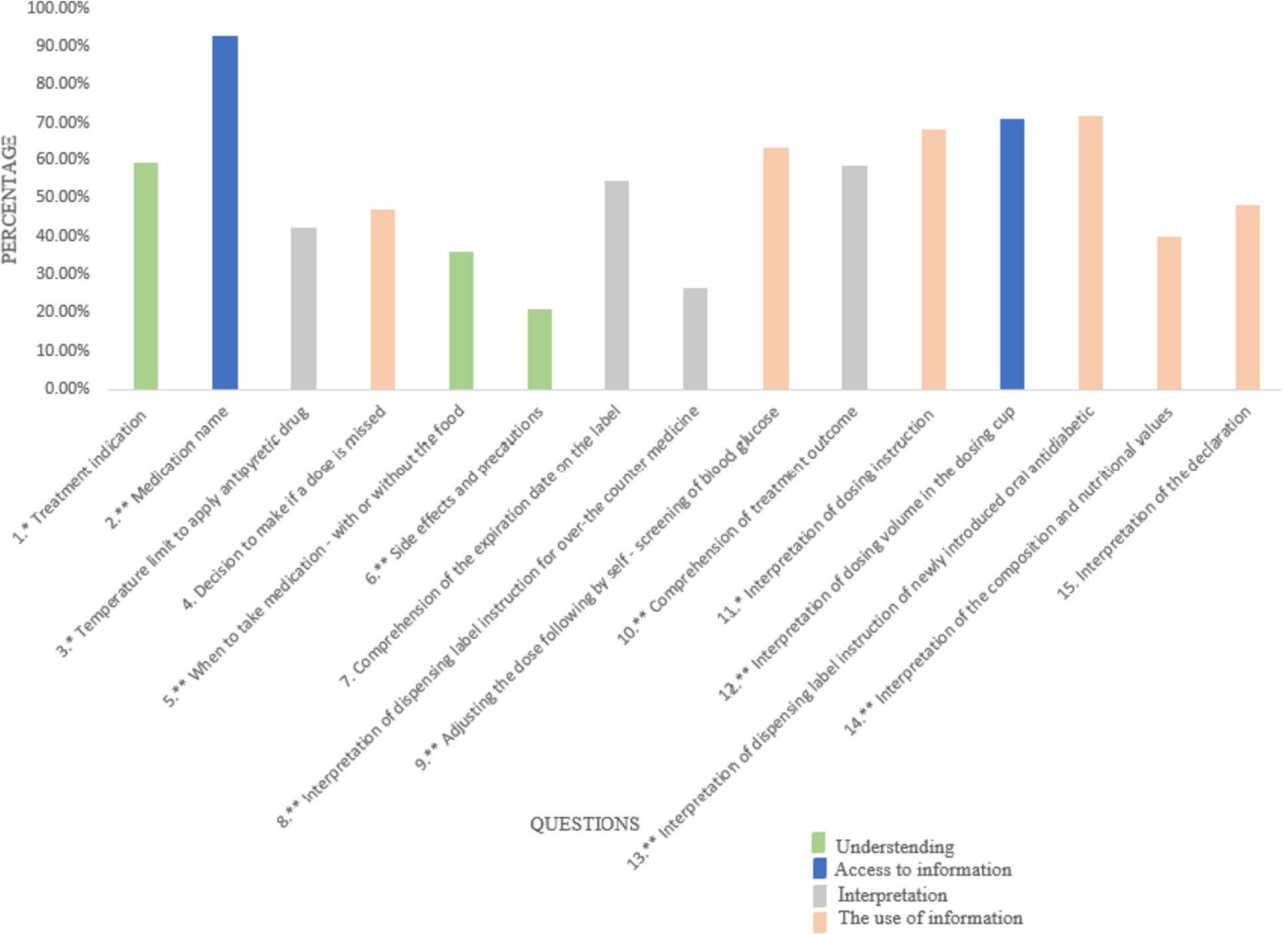
Distribution of correct answers (%) by questions in the final PTHL-DM instrument (*n* = 350)

The distribution of correct answers (%) for the final PTHL-DM instrument in DMT2 patients is shown in Fig. 2. More than 80% recognised the medicine, while less than 30% of respondents were aware of side effects and precautions and interpretation of dispensing label instructions for over-the-counter medicines.

For most questions, the difference between percentage of correct and wrong answers was statistically significant, except for questions 4, 7 and 15. Analysis of questions that reflect the extent of understanding domain in PTHL-DM showed that 21% of patients did not give the correct answer, 50% of participants gave one correct answer out of a total of 3. Regarding the domain Access to information, only 6% of participants had 1 correct answer, and 70% had both answers correct. The domain Interpretation showed 9% of participants had all incorrect answers, and 43% of them in the largest group had 2 correct answers, out of 4. The domain Use of information presented the least group of participants with all incorrect answers (3%) and the highest percentage of patients had 4 correct answers (28%), out of a total of 6.

#### Analysis of PTHL domains

The mean PTHL-DM total score was 7.8 ± 2.3, and scores for understanding, access to information, interpretation and use of information domains were 1.2, 1.6, 1.8 and 3.2, respectively. The range of correct answers for total PTHL-DM scores was from 3 to 13.

#### Analysis of levels of PTHL

When sociodemographic characteristics and low, medium and high levels of PTHL were analysed (Table 2), the results showed only 5% (*n* = 17) patients had high level of PTHL, 33.4% (*n* = 117) had medium and the rest were seen to have low PTHL level (62%, *n* = 216).

**Table 2:** Sociodemographic characteristics and health behaviours of DMT2 patients stratified by PTHL level

			**PTHL level**			
	**Characteristics**	**Total** **(*N* = 350)**	**Low** **(*N* = 216)**	**Medium** **(*N* = 117)**	**High** **(*N* = 17)**	χ2/p*
Gender, N (%)	Male	156 (44.6)	97 (62.2)	54 (34.6)	5 (3.2)	1.71/0.425
	Female	194 (55.4)	119 (61.3)	63 (32.5)	12 (6.2)	
Marital status, N (%)	Single	158 (45.1)	97 (61.4)	53 (33.6)	8 (5)	0.03/0.984
	Married/Common-law	188 (53.8)	117 (62.2)	62 (33.0)	9 (4.8)	
Children, N (%)	No	91 (26.0)	29 (31.9)	51 (56.0)	11 (12.1)	4.47/**0.001**
	One child	91 (26.0)	20 (22.0)	54 (59.3)	17 (18.7)	
	Two or more children	168 (48.0)	80 (47.6)	64 (38.1)	24 (14.3)	
Level of education, N (%)	Secondary school or less	138 (39.4)	96 (69.6)	41 (29.7)	1 (0.7)	11.23/**0.004**
	College/university/post-graduate	212 (60.6)	120 (56.6)	76 (35.8)	16 (7.5)	
Employment, N (%)	Employed	219 (62.6)	131 (59.8)	76 (34.7)	12 (5.5)	1.09/0.579
	Unemployed or Pensioner	131 (37.4)	85 (64.9)	41 (31.3)	5 (3.8)	
Monthly income per family member, N (%)	< 40,000 RSD	88 (25.1)	64 (72.7)	23 (26.1)	1 (1.1)	8.03/0.091
	40,000–60,000 RSD^a^	228 (65.1)	134 (58.8)	80 (35.1)	14 (6.1)	
	≥ 60,000 RSD	34 (9.8)	18 (52.9)	14 (41.2)	2 (5.9)	
HL^b^	Inadequate HL	116 (33.3)	72 (62.1)	40 (34.5)	4 (3.4)	9.19/0.057
	Marginal HL	222 (63.3)	141 (63.5)	70 (31.5)	11 (5)	
	Adequate HL	12 (3.4)	3 (25)	7 (58.3)	2 (16.7)	
Active exercise, N (%)	Never	57 (16.3)	42 (73.7)	14 (24.6)	1 (1.8)	12.66**/0.049**
	Less than once a week	135 (38.6)	79 (58.5)	50 (37)	6 (4.4)	
	1–2 times a week	118 (33.7)	78 (66.1)	34 (28.8)	6 (5.1)	
	3 or more times a week	40 (11.4)	17 (42.5)	19 (47.5)	4 (10)	
Smoking, N(%)	Smoker	178 (50.9)	125 (70.2)	48 (27)	5 (2.8)	11.90/**0.00**3
	Non-smoker	172 (49.1)	91 (52.9)	69 (40.1)	12 (7)	
Alcohol intake, N (%)	Never	156 (44.6)	84 (53.8)	59 (37.8)	13 (8.3)	12.57**/0.01**4
	Once a month	121 (34.5)	82 (67.8)	35 (28.9)	4 (3.3)	
	2 or more times a month	73 (20.9)	50 (68.5)	23 (31.5)	0 (0)	

*Abbreviation PTHL*, Pharmacotherapy literacy, *HL* Health literacy

^*^Bold *p* values denote statistical significance

^a^1RSD = 0.0085 EU

^b^Assessed with Serbian version of the Functional, Communicative and Critical Health Literacy Scale with 12 questions (FCCHL-SR12)

Higher ages are connected to low PTHL (*p* = 0.038). There was no statistical significance between PTHL level and HL score (*p* = 0.999), nor with years having diabetes (*p* = 0.249). The patients with one child were more prevalent in the group with high PHTL than those without children or with two and more children (*p* = 0.001). Patients with a low level of education (completed secondary school or less) were more prevalent in the group with low PHTL than their counterparts with higher education (*p* = 0.004).

With regards of HbA1c value, no statistical significance was found with PTHL level (χ2 = 3.03, *p* = 0.220).

Treatment regimen for DMT2 showed that the highest percentage of highly literate patients is in the group receiving insulin and oral medication (12%) in comparison with patients on oral medication, insulin only or diet (3.1%, 0% and 0%, respectively). Significant statistical difference was found with respect to treatment regimen and PTHL level (χ2 = 19.63, *p* = 0.003). A lower adequate literacy rate (low PTHL level) was observed for patients who take oral medications, then for those taking oral medication and insulin, diet and insulin only (65.3%, 54.7%, 50.0% and 22.2%, respectively).

Patients who have administrated a medication three or more times a day proved to have higher PTHL level (χ2 = 6.78, *p* = 0.034), than those taking the medicine once/twice a day.

The association of patients' access to health-related information and empowerment-related indicators with PTHL level is shown in Table 3. The results showed that low PTHL was least prevalent if the information was obtained from a pharmacist, in comparison to a doctor, internet or other sources (*p* < 0.001). The patients who are very interested in their health indicated higher PTHL (*p* < 0.001), as well as those who estimated their health status as good (*p* < 0.001).

**Table 3:** Access to empowerment-related indicators of the DMT2 patients stratified by PTHL level

			**PTHL level**			
	**Characteristics**	Total* (*N* = 350)	Low* (*N* = 216)	Medium* (*N* = 117)	High* (*N* = 17)	χ2/p*
Source of health information	Doctor	204 (58.3)	122 (59.8)	69 (33.8)	13 (6.4)	26.23/** < 0.001**
	Pharmacists	47 (13.4)	17 (36.2)	27 (57.4)	3 (6.4)	
	Internet	15 (4.3)	11 (73.3)	4 (26.7)	0 (0)	
	Other	84 (24.0)	66 (78.6)	17 (20.2)	1 (1.2)	
Interest in health	Not interested/Little	142 (40.6)	98 (69)	44 (31)	0 (0)	26.56/** < 0.001**
	Medium	172 (49.1)	102 (59.3)	60 (34.9)	10 (5.8)	
	Much or very interested	36 (10.3)	16 (44.4)	13 (36.1)	7 (19.4)	
Self-estimation of health status	Bad	99 (28.3)	57 (57.6)	41 (41.4)	1 (1)	20.57/** < 0.001**
	Good	201 (57.4)	117 (58.2)	68 (33.8)	16 (8.0)	
	Very good	50 (14.3)	42 (84)	8 (16)	0 (0)	

*Abbreviation PTHL* Pharmacotherapy literacy

^*^Bold *p* values denote statistical significance

### Analysis of predictors for low PTHL

#### Univariate and multivariate predictor models

Sociodemographic characteristics of participants (gender, marital status, children, education, employment, income), therapy, frequency of administration, health behaviors (exercise, alcohol intake, smoking), access to health-related information, and empowerment-related indicators (interest in health and self-estimation of health status) were used as predictors of low PTHL. Unadjusted odds ratios (OR) and 95% confidence intervals (CI) for factors associated with patients’ low PTHL were presented in Table 4. Predictors of low PTHL were higher age, lower education, lower income per family member, diet and OHAs used as a therapy, no active exercise, smoking status, alcohol intake, other sources of health information (information which were not received from doctors and pharmacists), little interest in health and bad estimation of health status.

**Table 4:** Sociodemographic characteristics as predictors for low PTHL

**Univariate analyses**	**OR**	**95% CI**	***p* value**
Age^a^	1.035	1.014–1.058	0.001
Education			
(College/university/post-graduate)	0.755	0.602–0.947	0.015
Monthly income per family member^a^	0.619	0.420–0.912	0.015
Therapy for DMT2 (Insulin/Insulin and OHA)	0.564	0.343–0.926	0.024
Active excercise^a^	0.770	0.603–0.984	0.037
Smoking (Non smoker)	0.770	0.603–0.984	0.037
Alcohol intake^a^	1.429	1.072–1.906	0.037
Source of health information^a^			
Pharmacists	0.381	0.197–0.735	0.004
Internet	1.848	0.569–6.004	0.307
Other	2.464	1.364–4.453	0.003
Interest in health^a^	0.763	0.625–0.931	0.008
Self-estimation of health status^a^	1.236	1.007–1.518	0.043
**Multivariate analyses**	**OR**	**95% CI**	***p* value**
Smoking (Non smoker)	0.784	0.616–0.997	0.048
Interest in health^a^	0.439	0.255–0.757	0.004
Source of health information^a^			
Pharmacists	0.301	0.151–0.601	0.001
Internet	0.760	0.258–2.242	0.619
Other	1.471	0.862–2.510	0.157
Self-estimation of health status^a^	0.367	0.156–0.863	0.021

^a^Age – continuous variable, Monthly income per family member – ordered variable (< 40,000 RSD, 40,000–60,000 RSD, > 60,000 RSD 1 to 3), Active exercise – ordered variable (never, less than once a week, 1–2 times a week, 3 and more times a week were coded from 1 to 4), Smoking – ordered variable (smoker and non-smoker were ordered from 1–3), Alcohol intake – ordered variable (never, once a month, 2 or more times a month were ordered from 1–3), Source of health information – information received from doctors represented reference group, information received from Pharmacists, Internet and Other are coded 1,2 and 3 respectively, Interest in health – ordered variable (Not interested/Little, Medium and Much and very interested were ordered from 1–4), Self-estimation of health status (Bad, Good and very good were ordered from 1–3). Other Multivariate analyses were performed with significant predictors from univariate analyses

Additionally, all significant predictors were included in multivariate analysis to assess independent predictors of low PTHL. Smoking was a significant independent predictor of low PTHL level. Little interest in health and assessment of health as bad were associated with a higher probability of low PTHL. The source of health information was also an independent predictor—a lower probability for low PTHL is seen if advice is received from a pharmacist compared to a doctor.

## Discussion

The concept of PTHL is mostly unknown for the majority of Serbian population. To the best of our knowledge, this is the first research to investigate levels of PTHL among the DMT2 patients in Serbia. Measuring PTHL for chronic non-communicable diseases, especially DM, is important but it is also important to use a condition (disease or content) specific instrument [48]. We adapted the existing self-administered, performance-based instrument and proved that the new one is specific for assessing DMT2 patients' medication literacy with satisfactory psychometric characteristics. The correlation between the initial questionnaire (PTHL-SR) and the adopted questionnaire (PTHL-DM) was relatively good. Also, the domains of these questionnaires were examined, with the best correlation with domain of understanding. Very good reliability was shown for 12 questions, one question showed good and two questions modest reliability. Similar findings were seen in the research conducted in Serbia [7]. Also, the demonstrated reliability and internal reliability through KR20, ICC coefficient and test–retest reliability test proved that the constructed PTHL-DM questionnaire is a reliable and validated instrument. Furthermore, we found that low level of PTHL was highly prevalent in DMT2 patients and identified that smoking habit, who are smokers, low interest in owns health and self/estimation of owns health as bad could individually predict low PTHL.

Diabetes prevalence (% of population ages 20 to 79) in Serbia was reported at 9.1% for 2021 [27, 30]. Diabetes prevalence grows with age, and it is estimated that almost a half of diabetic patients are over 65 years of age. In our sample patients over 65 years were represented with 40%. At the same time, the process of demographic ageing of the Serbian population manifests itself as a share of over 65 years of age is 21.3% [29]. Although diabetes is a major non-communicable and chronic condition that causes a significant degree of mortality and morbidity, to the best of our knowledge, no data is available for Serbia according to its prevalence by gender or levels of education. Some date is available regionaly, and by using data provided for the city of Belgrade we estimated the representativeness of study sample for the population of the elderly with DMT2 in Belgrade [28, 30].

Our foundings indicate the prevalence of patients with low PTHL level (62%).This was not in line with the research from Krajnovic et al., in Serbia [49], on the parents of pre-school children in which it was found that every tenth parent (10%) from rural areas and every fourth parent (25%) from urban areas had the highest PTHL level. Contrary to that, around half (51%) and one third (28%) of parents in rural and urban areas, respectively, had low PTHL levels. Having an elderly population perform the calculation with the mean-age of 62.5 years, who may have trouble reading and interpreting the questions, could offer an explanation for this. In the research conducted by Tefera et al. in Ethiopia [50], 17.3%, 26.3%, and 56.5% had low, medium, and high diabetic-related HL. This might be attributable to the variability of HL tools used, since the other tool measured informational, numeracy, and communicative HL relevant to diabetes. But also, the sociocultural and geographical variation might explain further differences.

According to the results, 21% of patients didn’t give any correct answer on the questions for understanding –most of them could not correctly explain the warning about exposure to the sun during therapy. Access to information was better, with 70% of patients having both correct answers. Interpretation/knowledge showed good results, with only 9% of patients having no correct answers, The questions on showing the target glycemic range and expiration date were correctly answered by 43% of patients. The use of information was with expected distribution, and questions related to the calculation of dose were correctly answered by most of the patients. In the research with Diabetes Numeracy test (DNT-15) the results showed that the problems faced by patients with DM include proper calculation of insulin dosage based on current blood glucose levels and carbohydrate intake [51]. These findings were not in line with our findings, but this difference may be due to several factors. Firstly, in Serbia there are some diabetic guidelines [52]. Secondly, different social and environmental factors can cause anxiety among the participants and increase the number of errors performed during the evaluation [53].

High PTHL level and proper medication adherence can contribute to achieving good glycemic control and preventing different complications among DMT2 patients [54].

Furthermore, 19 factors are investigated that can impact the level of PTHL in DMT2 patients. Association between PTHL and key factors from sociodemographic characteristics (ages, level of education, number of children),, health-related information (treatment regimen and frequency of drug administration), health behavior (alcohol intake, smoking and active exercise) and from empowerment-related indicators (source of health information, self-estimation of health and interest in health) was significant.

In this study, higher diabetic PTHL level is seen in males which is similar to finding in Tanzania [55] and Ethiopia [50]. However, no gender difference was found in these studies in achieving the targeted glycemic level. On the contrary, the researchers conducted in Japan [56] and Palestine [54] claimed that differences in gender can’t be explained by different body composition and that further investigation to examine efficacy/treatment response with regard to gender is needed.

Many studies in the past have shown that age and education were important factors associated with mediation level. Significant increase in medication literacy level was observed by aging and when academic level of the participants increased [4, 6, 8, 9, 12, 33]. We found that older patients have higher probability of low PTHL. Aging implies a higher prevalence of chronic pathologies and therefore an increase in medication but also it influences the ablility not only to have sufficient information, but to interpretate and to calculate doses. Also, less educated DMT 2 patients and with lowest monthly income had a higher likelihood of low PTHL. Our results are supported by studies conducted in Ethiopia and China [50, 57] where higher education attainment and higher household income were significantly associated with adequate literacy. A significantly low diabetic HL was also reported in illiterate patients than those who have a higher level of education in United Arab Emirates (UAE) [58] and Bangladesh [59].

In our study’s analysis, we found that patients who took medication three or more times a day, and those on insulin and OMAs proved to have higher PTHL level, which was expected as these patients have been exposed to a longer period of diabetes` education from the time of diagnosis. A similar finding was observed in the 2020 study conducted in the US [60]. A study by Singh et al. in India stated that patients receiving insulin therapy a significantly lesser score for interpreting prescription instructions when compared with those receiving only oral antidiabetics [61].

Alcohol intake, smoking and lack of physical activity were in direct correlation with low PTHL. In general, studies confirmed that changes in health behavior and weight loss can significantly reduce the risk of DMT2 [62].

Self-assessment of health status also represents another factor that affects PTHL [63–65]. The source of health information is a significant predictor of low PTHL, the research results showed that a probability of low PTHL decreses if the information is obtained from a pharmacist compared to a doctor, while the higher likelihood of low PTHL arises if the information is obtained from the internet or other source compared to a doctor.Slightly different findings were obtained in the research involvingparents of pre-school children, which showed the lowest probability of inadequate literacy when parents received information from doctors. One of the reasons why higher PTHL level is estimated in patients whose main source of information is a pharmacist is that pharmacists are one of the most accessible healthcare professionals. Pharmacists can counsel on monitoring glucose level, an appropriate diet and exercise routine and define the most appropriate hypoglycemic strategy for a certain patient [66]. Althogh DMT2 patients with one child were more prevalent in group with high PHTL than those without children or with two and more children (χ2 = 4.47, *p* = 0.001),we did not prove that number of children was a predictor of low PTHL level. Previous studies did not fully recognize this factor as important to investigate. We consider this factor important to include in our analysis as the safety of children is at risk due to parents’ medication illiterateness.

The DMT2 patients with low interest in health, and those who estimated their health as bad, had higher probability for low PTHL. This aligned with research findings involving parents of pre-school children conducted in Serbia [67] and other research [68], that showed those patients who rated their health as only fair or poor are twice as likely to have inadequate HL compared to those who rate their health as either good or excellent.

### Limitations

The scientific study ended up with some limitations, that are helpful for future investigations. The sample of this study used a convenient sample based on DMT2 patients selected from targeted healthcare institutions. Therefore, the study findings are limited to this sample, which could limit the generalisation of the results. Although diabetes is a major non-communicable and chronic condition that causes a significant degree of mortality and morbidity, to the best of our knowledge, no data on its prevalence by gender or levels of education is available in Serbia. Hence, our findings might not be generalized to overall Serbian DMT2 patients' population, we could prove some resemblance with the general population of Serbia. According to the Institute of Public Health of Serbia number of newly diagnosed cases of DMT2 in a Serbian population (0–75 + years) is higher in woman 52.3% [27]. There are more women than men (51.4% vs. 48.6%) in Serbia according to the Census in 2022 [28, 29]. In our sample woman are prevalent as well (55%) but this is not significant. The level of education in our sample was higher than that of the general Serb population; According to the 2022 census in Serbia, 16.4% of the population has college, higher and university education, and 53.1% secondary education.

Another limitation involved in this research survey reported the difficulty in instrument answering, since it is quite lengthy. The diabetes illiterate participants may encounter problems filling in the forms. The comprehensiveness of the research results can be augmented by further investigations across specific geographic regions and in various cultures.

## Conclusions

Among primary care patients with DMT2, low PTHL is independently associated with patients who are smokers, those with low interest in their health and patients who estimated their health as bad. Also, it is shown that patients who are on diet, OHAs or insulin only have higher probability for low PTHL than those on insulin and OHAs.

The current study revealed only the average number of diabetic populations have a medium PTHL level. Higher PTHL was reported in those patients who have one child, patients with the highest education, non-smokers, those who never consume alcohol and exercise 3 or more times a week. These patients are more likely to be highly literate with medications. Also, in Serbia a high percentage of DMT2 patients were found to have low PTHL.

Different patient empowerment programs and approaches aimed at raising PTHL would be essential in improving self-management and control of this widespread disease. Future research on a larger population in Serbia is necessary to draw conclusions about the levels of PTHL and their relationship with medication adherence and glycemic control.

### Supplementary Information

**Additional file 1.** STROBE Statement—Checklist of items that should be included in reports of *cross-sectional studies*

## Data Availability

The datasets used and/or analysed during the current study available from the corresponding author on reasonable request.

## Abbreviations

DMT2: Diabetes mellitus type 2
HL: Health literacy
PTHL: Pharmacotherapy literacy
OHA: Oral Hypoglycemic Agents
WHO: World Health Organization
