# Supplementary material for: Pharmacotherapy literacy level and predictors of low literacy among diabetes mellitus type 2 patients in Serbia
Source: BMC Public Health. 2023 Sep 19;23:1822. doi: 10.1186/s12889-023-16639-y (PMC10507974; doi:10.1186/s12889-023-16639-y)
Supplement: Supplementary file 1 — Additional file 1. STROBE Statement—Checklist of items that should be included in reports of cross-sectional studies [file 12889_2023_16639_MOESM1_ESM.doc]

STROBE Statement—Checklist of items that should be included in reports of ***cross-sectional studies***

|  | Item No | Recommendation |
| --- | --- | --- |
| **Title and abstract** | 1 | (*a*) Indicate the study’s design with a commonly used term in the title or the abstract  Cross-sectional study as stated in the Abstract. |
|  |  | (*b*) Provide in the abstract an informative and balanced summary of what was done and what was found  Provided in the Abstract. |
| Introduction | | |
| Background/rationale | 2 | Explain the scientific background and rationale for the investigation being reported  Included in the Background on pages 2-3. |
| Objectives | 3 | State specific objectives, including any prespecified hypotheses  Included in the Background on page 3. |
| Methods | | |
| Study design | 4 | Present key elements of study design early in the paper  Included in the Method on pages 3-6. |
| Setting | 5 | Describe the setting, locations, and relevant dates, including periods of recruitment, exposure, follow-up, and data collection  Included in the Method, on pages 3-6. |
| Participants | 6 | (*a*) Give the eligibility criteria, and the sources and methods of selection of participants  Included in the Method, Sample and data collections, on page 3. |
| Variables | 7 | Clearly define all outcomes, exposures, predictors, potential confounders, and effect modifiers. Give diagnostic criteria, if applicable  Included in the Method, on pages 3-6. |
| Data sources/ measurement | 8* | For each variable of interest, give sources of data and details of methods of assessment (measurement). Describe comparability of assessment methods if there is more than one group  Included in the Method, on pages 5. |
| Bias | 9 | Describe any efforts to address potential sources of bias  Included on pages.3 and 11. |
| Study size | 10 | Explain how the study size was arrived at  Included in the Method, on pages 4 and 5. |
| Quantitative variables | 11 | Explain how quantitative variables were handled in the analyses. If applicable, describe which groupings were chosen and why  Included in the Method, on pages 3-6. |
| Statistical methods | 12 | (*a*) Describe all statistical methods, including those used to control for confounding  Included in the Method, on pages 5and 6. |
|  |  | (*b*) Describe any methods used to examine subgroups and interactions  Not applicable. |
|  |  | (*c*) Explain how missing data were addressed |
|  |  | (*d*) If applicable, describe analytical methods taking account of sampling strategy |
|  |  | (*e*) Describe any sensitivity analyses  Not applicable. |
| Results | | |
| Participants | 13* | (a) Report numbers of individuals at each stage of study—eg numbers potentially eligible, examined for eligibility, confirmed eligible, included in the study, completing follow-up, and analysed  Included in the Methods on page 3 and in the Results on pages6 and 7 and in the Discussion on pages 10 and 11.. |
|  |  | (b) Give reasons for non-participation at each stage  Not applicable. |
|  |  | (c) Consider use of a flow diagram  Not required. |
| Descriptive data | 14* | (a) Give characteristics of study participants (eg demographic, clinical, social) and information on exposures and potential confounders  Included in the Results on page9. |
|  |  | (b) Indicate number of participants with missing data for each variable of interest |
| Outcome data | 15* | Report numbers of outcome events or summary measures |
| Main results | 16 | (*a*) Give unadjusted estimates and, if applicable, confounder-adjusted estimates and their precision (eg, 95% confidence interval). Make clear which confounders were adjusted for and why they were included  Included in the Results on pages 9 and 10. |
|  |  | (*b*) Report category boundaries when continuous variables were categorized  Included in the Results on pages6-9. |
|  |  | (*c*) If relevant, consider translating estimates of relative risk into absolute risk for a meaningful time period  Not applicable. |
| Other analyses | 17 | Report other analyses done—eg analyses of subgroups and interactions, and sensitivity analyses  . |
| Discussion | | |
| Key results | 18 | Summarise key results with reference to study objectives  Included in the Discussion on pages 9. |
| Limitations | 19 | Discuss limitations of the study, taking into account sources of potential bias or imprecision. Discuss both direction and magnitude of any potential bias  Included in the Discussion, Limitations on page 12. |
| Interpretation | 20 | Give a cautious overall interpretation of results considering objectives, limitations, multiplicity of analyses, results from similar studies, and other relevant evidence  Included in the Discussion on pages 9-12. |
| Generalisability | 21 | Discuss the generalisability (external validity) of the study results  Included in the Conclusion on page 12. |
| Other information | | |
| Funding | 22 | Give the source of funding and the role of the funders for the present study and, if applicable, for the original study on which the present article is based  Included on page 12. |

*Give information separately for exposed and unexposed groups.

**Note:** An Explanation and Elaboration article discusses each checklist item and gives methodological background and published examples of transparent reporting. The STROBE checklist is best used in conjunction with this article (freely available on the Web sites of PLoS Medicine at http://www.plosmedicine.org/, Annals of Internal Medicine at http://www.annals.org/, and Epidemiology at http://www.epidem.com/). Information on the STROBE Initiative is available at www.strobe-statement.org.
